# Self-referral practice and associated factors among women who gave birth in South Gondar zone primary hospitals, Northwest Ethiopia: a cross-sectional study design

**DOI:** 10.3389/fpubh.2023.1128845

**Published:** 2023-06-05

**Authors:** Ayenew Eshetie, Tadele Biresaw Belachew, Wubshet Debebe Negash, Desale Bihonegn Asmamaw, Sualiha Abdulkader Muktar, Adane Kebede

**Affiliations:** ^1^Department of Health Systems and Policy, College of Medicine and Health Sciences, Institute of Public Health, University of Gondar, Gondar, Ethiopia; ^2^Department of Reproductive Health, College of Medicine and Health Sciences, Institute of Public Health, University of Gondar, Gondar, Ethiopia; ^3^International Institute for PHC Program Coordinator, Addis Ababa, Ethiopia

**Keywords:** self-referral practice, primary hospital, South Gondar, Ethiopia, factors

## Abstract

**Background:**

Patient self-referral is when patients refer themselves to upper-level health facilities without having to see anyone else first or without being told to refer themselves by another health professional. Self-referral leads to a diminished quality of healthcare services. However, globally, many women who gave birth referred themselves to hospitals without having referral sheets, including in Ethiopia and the study area. Therefore, this study aimed to assess self-referral practice and associated factors among women who gave birth in South Gondar zone primary hospitals in Northwest Ethiopia.

**Methods:**

A cross-sectional mixed-method study was conducted among women who gave birth in South Gondar zone primary hospitals between 1 June 2022 and 15 July 2022. Semi-structured questionnaires were used to gather quantitative data from 561 participants who were selected by a systematic random sampling technique. Interview guides were used to collect qualitative data from selected six key informants. Quantitative data were entered into Epi Data version 4.6.0.4 and then exported to the statistical software SPSS version 25 for further analysis. Thematic analysis using open code version 4.02 software was applied for qualitative data analysis. A binary logistic regression analysis was employed. In a bivariable analysis, a *p* < 0.25 was used to select candidate variables for multivariable analysis. *P* < 0.05 and a 95% confidence interval were used to determine significant variables on the outcome of interest.

**Results:**

The overall magnitude of self-referral was 45.6%, with 95% CI (41.5%, 49.9%). They had no antenatal care (ANC) follow-up (AOR = 3.02, 95% CI: 1.64–5.57) and 1–3 ANC follow-ups (AOR = 1.57, 95% CI: 1.03–2.41), poor knowledge about the referral system (AOR = 4.04, 95% CI: 2.30–7.09), and use of public transportation (AOR = 2.34, 95% CI: 1.43–3.82), which were significantly associated with self-referral practice.

**Conclusion:**

This study showed that nearly half of the deliveries were self-referred. ANC follow-up, women's knowledge of the referral system, and mode of transportation were factors significantly associated with the self-referral practice. Therefore, developing awareness-creation strategies and increasing coverage of ANC 4 and above are necessary interventions to reduce the self-referral practice.

## Background

A referral system allows patients to access care in health centers (HCs) before higher levels of care, such as second- and third-level hospitals, are offered (1). A referral is a process by which a health professional temporarily or permanently transfers responsibility for care to another health professional, social worker, or community due to their inability or limitation to provide the necessary care (2). In a referral system, patients are transferred between medical centers with or without a referral sheet, which are the so-called self-referrals (3). A patient self-referral is a situation when patients refer themselves to upper-level health facilities without having to see anyone else first or without being told to approach another health professional (4). Alternative terms have also been employed by further studies, such as bypassing the primary-level facilities. Nonetheless, the overall meaning behind these terms remains the same (5).

The extent of self-referral to higher-level hospitals is alarming worldwide. However, based on symptoms, most of the disease presentations could have been managed adequately in a primary healthcare unit (PHCU) setting (6). Developing nations, including Ethiopia, have referral policies, but putting those into practice is complex (7–9). In many Asian and African countries, more than two-thirds of patients bypassed outpatient departments at lower-level health facilities (6, 10–12). In Africa, particularly Sub-Saharan African countries, 19.35–47.2% of women bypassed PHCUs for maternal–child healthcare (13–17). As a result, hospitals were overcrowded with patients who could be treated in lower-level facilities (18).

Bypassing HCs has deleterious effects on the healthcare system, particularly on healthcare services for the general population (3). It wastes the time of highly qualified medical personnel on minor cases and overstretched human and physical resources. As a result, there are delays in effective management, poorer patient outcomes, declining quality of care, and increased dissatisfaction among providers and healthcare users (19). Bypassing HCs leads to underutilization of the HCs and overburdening of primary, general, and specialized hospitals (6, 20). From the patients' or clients' side, bypassing causes extra costs in terms of transportation, living, and medical expenses (3, 16, 17, 21). Patients are also vulnerable to drug-resistant bacteria linked with hospital-associated healthcare infections (22).

The significant factors that influence self-referral practice were socio-demographic (age, education, residence, income, and good knowledge) (10, 12, 14, 17, 23–26), obstetric (primigravida, obstetric complication, primiparity, and ANC visit) (17, 24, 26, 27), access, perceived privacy and confidentiality of services, having no equipment, enrolled community-based health insurance, health facility, service quality, and history of visiting a hospital (4, 16, 17, 24, 27).

The Ethiopian health service operates a three-tier structure of PHCUs, general hospitals, and specialized hospitals (2). The catchment population and the package of care provided at each level are different, but all are interlinked (2). PHCUs are the first level of contact of individuals with the national health system that brings maternal and child care as close as possible to where people live and work (28). In Ethiopia, HCs serve as referral centers for health posts (HPs), and primary hospitals serve as referral centers for HCs (29). General and specialized hospitals are meant to handle sophisticated cases (3).

The Ethiopian health policy has strategies for developing a referral system (30). These strategies are improving accessibility, optimizing utilization, rationalizing costs, assuring continuity, improving quality of care at all levels, and strengthening communication within the healthcare system (30). The referral system ensures that every level of the referral chain can withstand the expected functions (28). It also enables greater efficiency and less burden on hospitals (31). Pre-referral stabilization and enhanced communication can avert a considerable number of deaths (32).

The Ministry of Health in Ethiopia has developed different referral implementation guidelines, referral formats, and feedback mechanisms. In addition, there are 24-h referral services and liaison nurses in health facilities to strengthen the referral system (2, 3, 29, 33). Ethiopia also invests a large amount of money in HCs and HPs. According to Ethiopia's 2017 National Health Account Report, more than one-third of total health expenditure goes to HCs and HPs (34). Despite this, the community usually seeks care directly from hospitals without an official referral from a HC or HP and without looking for any prior source of care (1, 3, 35). Even though the extent of self-referral is not well documented and reported in the study area for childbirth, it accounts for nearly 63.9% of outpatients (25).

There is limited evidence concerning self-referral practice in Ethiopia and the study area. The available literature in Ethiopia concerning self-referral practice recommends conducting a mixed-method study (24). Additionally, the available literature in Ethiopia did not include important factors such as household wealth, knowledge of the referral system, cleanliness, privacy and confidentiality, and timely attention at HCs. Finally, evidence generated from this study could help policymakers, facility managers, and healthcare providers in planning interventions to reduce self-referral practice. Therefore, this study aimed to assess self-referral practice and associated factors among women who gave birth in South Gondar zone primary hospitals, North West Ethiopia.

## Methods

### Study area and period

A cross-sectional mixed-method study was conducted among women who gave birth in South Gondar zone primary hospitals between 1 June 2022 and 15 July 2022. The South Gondar zone is located in the northwest part of Ethiopia. The town of the South Gondar zone is Debre Tabor, which is 654 km from Addis Ababa, the capital city of Ethiopia, and 108 km from Bahir Dar, the capital city of Amhara regional state. According to the South Gondar zone city administration report, this zone is organized into seven town administrations and 13 districts. Additionally, there were 9 hospitals, 109 HCs, and 402 HPs in this zone at the time of data collection. Of the nine hospitals, one is a specialized hospital, and the other eight are primary hospitals. Based on the 2021 Amhara regional state population estimation, this zone has a total population of 2,651,350, of which 1,320,659 are women, 536,368 are non-pregnant women aged 15–49 years, and 89,351 are the estimated numbers of pregnant women.

### Study population

The study population comprised women who gave birth in South Gondar zone primary hospitals. As part of the qualitative component, we selected health professionals who worked in hospitals and had access to information. Women who had emergency cases and were unable to respond, those who were critically ill, health professionals who were on annual leave, and those who were ill during the period of data collection were excluded.

### Sample size and sample size procedure

**Figure 1:**
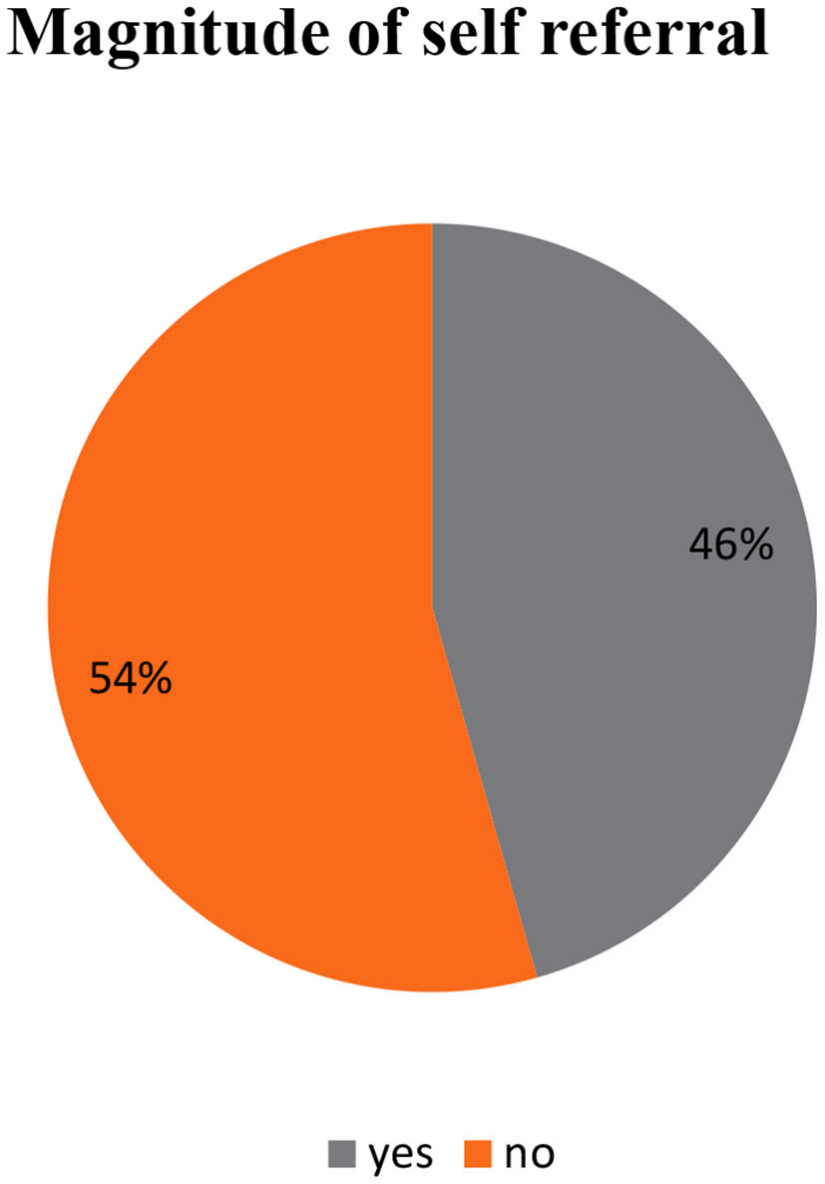
Diagrammatic representation of sampling procedure of women who gave birth in South Gondar zone primary hospitals, Northwest, Ethiopia, 2022. PH, Primary Hospitals; ABPH, Andabet PH; AZPH, Adiss zemene PH & Dr. AMPH=Dr. Ambachew memorial PH; EPH, Ebenate PH, MEPH, Mekane Eyesuse PH; MKMPH, Megbaru Kebede Memorial PH; NMPH, Nefase Mecha PH.

The sample size (for the first objective) was determined using a single population proportion formula by considering a 95% confidence level (zα/2 = 1.96), 5% margin of error, and 67% proportion of self-referral in Nigist Eleni Memorial Hospital, South Ethiopia (24), with 10% non-response rate, and the final sample size was 584 (Figure 1). A systematic random sampling technique was used with a K-value of 2. We have used an average monthly report on the total number of mothers who attended the facility, which was 1,320 deliveries per month. On delivery registration lists, the first study participant was determined randomly. A purposive criterion (information-rich about the outcome, had experienced, and indicated by other samples) based sampling technique was used for the qualitative component.

### Study variables

#### Dependent variable

For this study, self-referral is defined as women who bypassed the nearby HC and presented themselves at primary hospitals for childbirth without a previous history of appointment and ANC follow-up classified as “Yes” or “No” (13, 15, 25, 36).

#### Independent variable

The independent variables were socio-demographic factors (age, residence, wealth index, and educational and occupational status), obstetric factors (gravidity, parity, ANC follow-up, previous and current pregnancy complications, and previous childbirth complications), health facility and service provision-related factors (timely attention at HC, cleanliness at HC, confidentiality at HC, history of visiting hospitals, and knowledge of the referral system), and access to health service-related factors (distance to hospital, access to transportation, mode of transportation to the hospital, and HC location).

**Figure 2:**
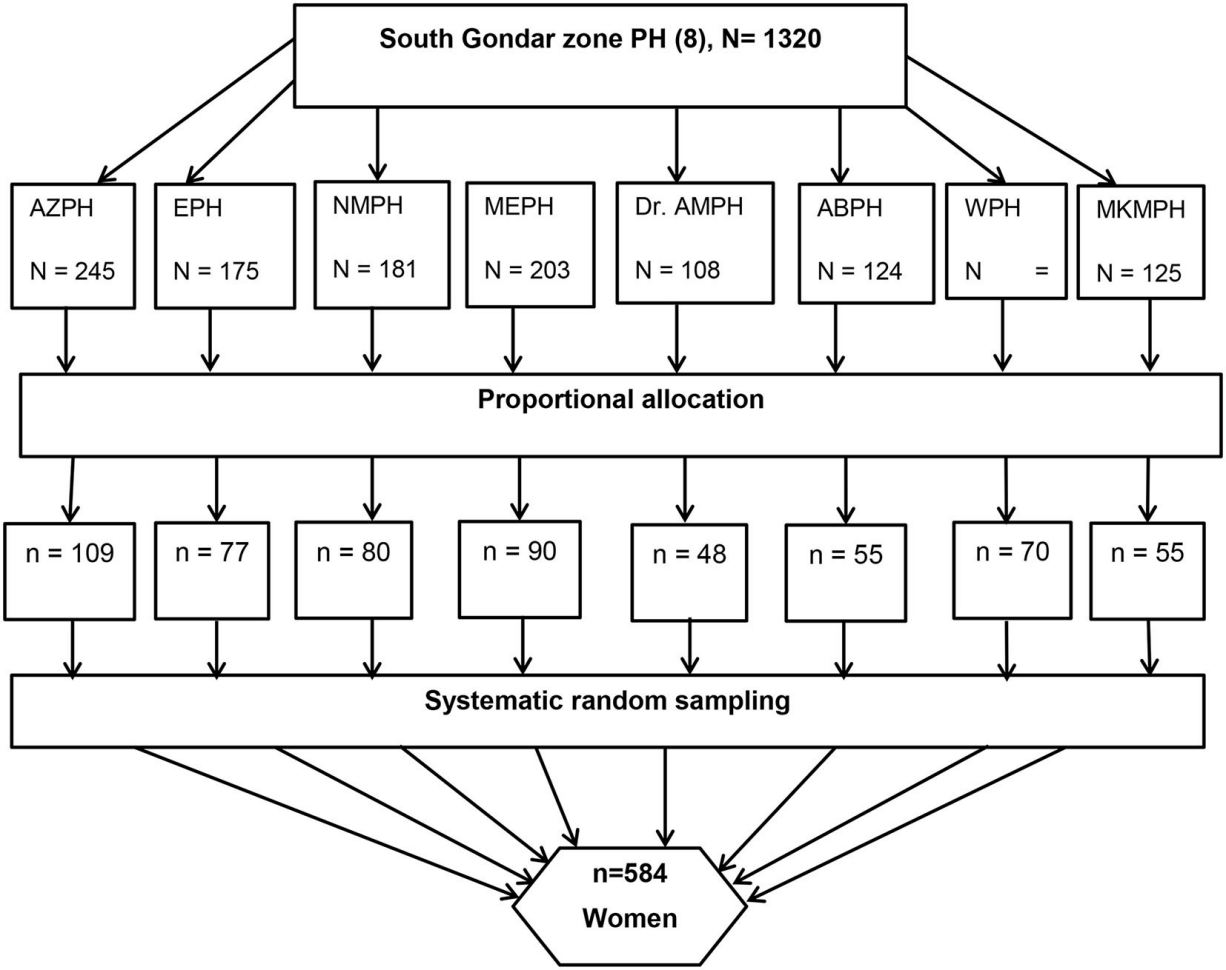
Conceptual framework developed from different pieces of literature.

The conceptual framework summarized different factors to assist in exploring factors associated with self-referral practice from different works of literature, including socio-demographic characteristics, health facility and service provision-related factors, health service accessibility-related factors, and obstetric factors. The framework was adapted from different pieces of literature (4, 10, 15, 17, 20, 23–25, 37, 38) (Figure 2).

#### Wealth index

All asset variables were coded into binary variables. All the assumptions of principal component analysis (PCA) were checked. The first PCA was generated from a group of 21 variables. Based on the first PCA, an aggregated score was calculated for each of the surveyed households, which was grouped into quintiles, with quintile 1 representing the “poorest” and quintile 5 representing the “richest” (13, 25, 39).

#### Knowledge of the referral system

Eight questions were used to measure the knowledge of women about the referral system. If the woman answers seven to eight questions, she is considered to have good knowledge; if she answers four to six questions, she is considered to have fair knowledge; if answers less than four questions, she has poor knowledge (25, 38).

#### Childbirth and pregnancy complications

A mother reported at least one of these as follows: vaginal bleeding, severe headache, blurred vision, abnormal body movement, malpresentation, malposition, intrauterine fetal death, prolonged labor, and leakage of amniotic fluid (40).

#### Distance

This was measured based on women's self-reported time to reach the hospital on foot in minutes. Then, the time was changed into hours and categorized into three (1 = “ <1 h”, 2 = “1–2 h”, and 3 = “more than 2 h”) (32).

#### Data collection tools and procedure

The quantitative, semi-structured interviewer-administered questionnaire was used which was adapted from different pieces of literature (16, 24, 33, 38). First, the questionnaire was prepared in English and then translated into the local language, Amharic, and back to English to check consistency by an independent translator (41, 42); for the qualitative data, an interview guide was used. KII was used as a qualitative data collection technique with permission from the participants. The interview proceedings were audio-taped.

#### Data quality control and assurance

For the quantitative part, 1-day training was given to data collectors and supervisors before data collection on the objectives, the importance of the study, confidentiality issues, participants' rights, informed consent, and techniques of interviewing by the principal investigator. Eight nurses with BSc degrees and two supervisors (BSc) who have skills in supervision participated in the data collection process. A pretest was done by the principal investigator on 30 postpartum women (5%) at Kolla Deba primary hospital, which is found in the neighboring central Gondar zone. Findings and experiences from the pretest were utilized in modifying the data collection tool. The data collectors were proficient in the Amharic language, and they did not belong to SGZPH.

The principal investigator and supervisors supervised data collectors and the whole process in the delivery ward. The data collectors, supervisors, and principal investigator manually checked the field questionnaire for completeness and consistency daily. Problems faced were discussed, and timely solutions were taken daily. Finally, the completeness of the questionnaire was checked and manually cleaned before data entry. Qualitative analyses were performed manually using qualitative content analysis. This process included open coding, abstracting, and creating classes. All individual interviews were transcribed, and the texts were read several times. Larger themes were formed by grouping the categories. Lastly, the quantitative and qualitative components were compared, synthesized, and discussed.

### Data processing and analysis

The questionnaire was coded uniquely by the principal investigator and supervisors, and then, double entry into EpiData version 4.6.0.4 software was done to minimize data entry errors. The quantitative data, entered into Epi Data version 4.6.0.4, were exported to SPSS version 25 statistical software for coding and further analysis. Tables, graphs, and texts were used to present descriptive statistics such as frequency, percentage, median, and interquartile range (IQR). Normality tests such as histogram, skewness, and kurtosis were employed to see the normal distribution of continuous independent variables and to identify which summary measure is appropriate to use. Accordingly, the distribution was found to be asymmetric. All continuous independent variables were categorized. The chi-square (χ^2^) assumption was checked for all independent variables, and all variables passed except the marital and occupational statuses of the woman. Multicollinearity among independent variables was checked using the variance inflation factor (VIF) and was found less than five multicollinearities (mean value = 1.5). A binary logistic regression analysis was employed to see the relationship between dependent and independent variables and to select the candidate variables for multivariable analyses. Independent variables with a *p* < 0.25 during bivariable logistic regression analyses were entered into the multivariable logistic regression analyses to control for confounding factors. Finally, an adjusted odds ratio (AOR) with 95% CI and a *P* < 0.05 was used to ascertain factors associated with self-referral. Model fitness was tested using the Hosmer–Lemeshow goodness-of-fit test (*p* = 0.84).

The interviews with key informants were properly recorded, and the audio recordings were transcribed into the local language and translated into English by the principal investigator. The translated qualitative data were entered into Open Code software version 4.02 after being converted to plain text. Thematic analysis was used to analyze the data. Subsequently, the codes were organized under six main themes. These themes were lack of skilled providers, medical equipment and drugs, trust, privacy at HC, better service at a hospital, and behavioral factors. The qualitative and quantitative data were collected and analyzed separately. Finally, they were integrated during the interpretation of the findings.

### Ethical consideration

Ethical clearance was obtained from the Institutional Review Board (IRB) of the University of Gondar, College of Medicine and Health Sciences, Institute of Public Health (Ref. No. IPH/2119/2014). A letter of cooperation from the hospital administrators was written to obstetric ward coordinators. Written informed consent was taken from all of the study participants after being informed of the study objectives, expected outcomes, benefits, and risks associated with it. Confidentiality of responses was maintained throughout the study.

## Results

### Sociodemographic characteristics of the participants

A total of 561 women participated in this study, with a response rate of 96%. Nearly half (49.7%) of participants were in the age group of 25–34 years, with a median age of 27 years (IQR: 23, 31 years). More than half (52.4%) of the participants were urban residents. A total of 218 participants (38.9%) had not attended formal education, and 180 (32.1%) and 107 (19.1%) participants were from the poorest and richest family wealth index, respectively (Table 1).

**Table 1:** Socio-demographic characteristics of the participants in South Gondar zone primary hospitals, Northwest, Ethiopia, 2022 (*n* = 561).

**Variables**	**Categories**	**Frequency**	**Percentage**
Age in years	16–24	191	34.0
	25–34	279	49.7
	35–45	91	16.2
Residence	Urban	293	52.2
	Rural	268	47.8
Marital status	Married	543	96.8
	Single	6	1.1
	Divorced	12	2.1
Wealth index	Poorest	180	32.1
	Poorer	51	9.1
	Middle	123	21.9
	Richer	100	17.8
	Richest	107	19.1
Women education	No formal education	218	38.9
	Primary (1–8)	128	22.9
	Secondary (19–12)	110	19.6
	More than secondary	105	18.7
Women occupation	Government employee	66	11.8
	Housewife	424	75.6
	Merchant	22	3.9
	Student	35	6.2
	Others^*^	14	2.5
Husband education (*n =* 543)	No formal education	211	38.9
	Primary (1–8)	105	19.3
	Secondary (9–12)	83	15.3
	More than secondary	144	26.5
Husband occupation (= 543)	Farmer	303	55.8
	Government employee	126	23.2
	Merchant	76	14.0
	Other^**^	38	7.0

* Daily laborer and private employee

** student, daily laborer, private employee, and driver.

A total of six participants took part in the qualitative part of the study. The age ranged between 29 and 36 years. All of the participants were men. More than half (4/6) of the participants had worked for more than 6 years (Table 2).

**Table 2:** Background information of the KII participants in South Gondar zone primary hospitals, Northwest, Ethiopia, 2022.

**Code**	**Sex**	**Age**	**Profession**	**Position**	**Experience years**
01	M	29	BSC midwife	MCH head	8
02	M	32	Dr.	Medical director	6
03	M	33	BSc nurse	CEO	10
04	M	36	IESO	IESO	12
05	M	34	BSC nurse	Liaison officer	10
06	M	32	BSC midwife	MCH head	6

### Obstetric characteristics of participants

Among 561 participants, two-thirds (66.1%) were multigravida and nearly two-thirds (63.6%) were multiparous. Among 357 participants, 109 (30.5%) had a history of complications during delivery. Concerning ANC, 257 (45.5%) had reported that they had 4 or more ANC follow-ups (Table 3).

**Table 3:** Obstetric characteristics of study women in South Gondar zone primary hospitals, Northwest, Ethiopia, 2022 (*n* = 561).

**Variables**	**Frequency**	**Percentage**
**Gravidity**		
Primigravida	190	33.9
Multigravida	371	66.1
**Previous pregnancy complication (*****n** =* **372)**		
Proportion with no previous pregnancy	242	65.1
Proportion with previous pregnancy	130	34.9
**Parity**		
Primiparous	204	36.4
Multiparous	357	63.6
**Previous child birth complication (*****n** =* **357)**		
Proportion with no previous child complication	248	69.5
Proportion with previous child complication	109	30.5
**ANC attendance**		
No ANC follow up	88	15.7
1–3 follow up	216	38.8
≥ 4	257	45.5
**Current pregnancy complications**		
Proportion with no current pregnancy complication	415	74.0
Proportion with current pregnancy complication	146	26.0

### Health service access-related Factors

A total of 155 (27.6%) participants estimated that they take more than 2 h to reach the hospital. The median time to reach a hospital was 1.5 h (IQR: 0.5, 2.5 h). More than two-thirds (79.1%) of the participants used public transportation when they went to the hospital (Table 4).

**Table 4:** Access-related factors of study participants in South Gondar zone primary hospitals, Northwest, Ethiopia, 2022 (*n* = 561).

**Variables**	**Categories**	**Frequency**	**Percentage**
Distance to hospital in an hour on foot	<1 h	175	31.2
	1–2 h	231	41.2
	>2 h	155	27.6
Access to transportation	Proportion with no access to transportation	64	11.4
	Proportion with access to transportation	497	88.6
Mode of transportation	Foot	117	20.9
	Public (private)	444	79.1
Health center location Place of ANC visit (*n =* 473)	Inconvenient	204	36.4
	Convenient	357	63.6
	Hospital	197	41.6
	Health center	246	52.0
	Other^*^	30	6.3

^*^Health post and private clinic.

### Individual factors

A total of 137 (24.4%) and 170 (30.3%) women had good and poor knowledge of the referral system, respectively. Two-thirds (66%) of the participants had a history of visiting the hospital (Table 5).

**Table 5:** Individual factors of study women in South Gondar zone primary hospitals, Northwest, Ethiopia, 2022 (*n* = 561).

**Variables**	**Categories**	**Frequency**	**Percentage**
Knowledge of the referral system	Poor	170	30.3
	Fair	254	45.3
	Good	137	24.4
History of visiting hospital	Proportion with no history of visiting hospital	191	34.0
	Proportion of with history of vising hospital	370	66.0

The overall magnitude of self-referral practice in South Gondar zone primary hospitals was 46% with 95% CI (41.5%, 49.9%) (Figure 2).

### Reasons for self-referrals for their delivery decision

The most popular reason for self-referral practice was the family intention for quality delivery (33.6%), followed by a lack of trust in the health center (17.6%). On the other hand, no respect for full-care providers appeared to be the least common reason (9.8%) (Table 6).

**Table 6:** Reasons for self-referral practice among women who gave birth in South Gondar zone primary hospitals Northwest, Ethiopia, 2022 (*n* = 256)^a^.

**Reasons for self-referral to hospital**	**Frequency**	**Percentage**
Family intention for quality delivery	86	33.6
Lack of trust at health center	45	17.6
No operation at health center	36	14.1
lack of equipment and drug	29	11.3
Better service at hospital	25	9.8
No respect full care providers	24	9.4
No skilled care providers in the health center	21	8.2

^a^Multiple responses.

### Factors associated with the self-referral practice

In the final multivariable binary logistic regression analysis, ANC follow-up, knowledge of the referral system, and mode of transportation to a hospital were significantly associated with the dependent variable at a *p* < 0.05.

Accordingly, the odds of self-referral practice among women who had no ANC follow-up and 1–3 ANC follow-ups were 3.02 (AOR = 3.02, 95% CI: 1.64–5.57) and 1.57 times (AOR = 1.57, 95% CI: 1.03–2.41) higher compared to those who had four and more ANC follow-ups, respectively. This finding was supported by the qualitative findings as half of the participants reported: “*According to our previous assessment at our hospital, mothers who came without a referral from a health center or came on their own will didn't meet [WHO] recommended antenatal care.” (36-year-old male IESO)*.

“*Most mothers have [ANC] follow up but it is below the standard. Most have at least one or two [ANC] follow up. Almost 90% and above have one or two follow up, but there is a problem regarding 4 and more [ANC] follow up.” (33-year-old male CEO)*.

The odds of self-referral practice among women who had poor and fair knowledge of the referral system were 4.04 times (AOR = 4.04, 95% CI: 2.30–7.09) and 3.07 times (AOR = 4.04, 95% CI: 1.84–5.13) higher compared to those who had good knowledge, respectively. This result was also supported by the qualitative part as most of the participants said: “*Awareness creation about the referral system is created during antenatal care visits and conferences but it is not too detailed and enough. It is not to the level that going to hospital is extravagant. It is also interrupted. Therefore self-referral is a problem of poor awareness and service interruption.”[SIC] (33-year-old male CEO)*.

The finding was also supported by the KI interview from *the liaison* office.

“*When we discussed with mothers under the presence of health extension workers, they did not know the whole structure of the health system and the referral system. Even if there is a health center in their area, they come straight to our hospital whenever they want and they often have less understanding when we evaluate.” (34-year-old liaison officer)*.

Moreover, the odds of self-referral practice among women who used public transportation were 2.34 times (AOR = 2.34, 95% CI: 1.43–3.82) higher compared to those who travel on foot (Table 7).

**Table 7:** Bivariable and multivariable analyses of potential factors associated with self-referral practice among women who gave birth in South Gondar zone primary hospitals, Northwest, Ethiopia, 2022.

**Variables**	**Self-referra**		**COR (95%CI)**	**AOR (95%CI)**
	**No**	**Yes**		
**Age**				
35–45	43	48	1	1
16–24	105	86	0.73 (0.44–1.21)	1.28 (0.63–2.61)
25–34	157	122	0.70 (0.43–1.12	0.94 (0.55–1.61)
**Residence**				
Rural	130	138	1	1
Urban	175	118	0.64 (0.46–0.89)	1.36 (0.80–2.31)
**Wealth index**				
Poorest	102	78	1	1
Poorer	28	23	1.074 (0.575–2.008)	0.86 (0.44–1.72)
Middle	60	63	1.373 (0.867–2.176)	1.56 (0.93–2.61)
Richer	58	42	0.947 (0.577–1.553)	1.11 (0.65–1.91)
Richest	57	50	1.147 (0.709–1.855)	1.16 (0.67–2.00)
**Women educational status**				
No formal education	97	121	1	1
Primary (1–8)	73	55	0.604 (0.389–0.938)	0.60 (0.35–1.03)
Secondary (1–12)	62	48	0.621 (0.391–0.985)	0.78 (0.40–1.49)
More than secondary	73	32	0.351 (0.214–0.576)	0.59 (0.29–1.17)
**Knowledge of referral system**				
Good	103	34	1	1
Poor	73	97	4.03 (2.46–6.59)	4.04 (2.30–7.09)^***^
Fair	129	125	2.94 (1.86–4.65)	3.07 (1.84–5.13)^***^
**Gravidity**				
Multigravida	195	176	1	1
Primigravida	110	80	0.81 (0.57–1.15)	0.83 (0.49–1.42)
**ANC follow up**				
≥4	166	91	1	1
No ANC follow up	28	60	3.91 (2.33–6.55)	3.02 (1.64–5.57)^***^
1–3 ANC follow up	111	105	1.73 (1.19–2.50)	1.57 (1.03–2.41)^*^
**Location of health center**				
Convenient	201	156	1	1
Inconvenient	104	100	1.24 (0.88–1.75)	1.19 (0.79–1.78)
**Mode of transport to hospital**				
Foot	80	37	1	1
Public (private)	225	219	2.11 (1.37–3.24)	2.34 (1.43–3.82)^**^
**History of visiting hospital**				
No	84	107	1	1
Yes	221	149	0.53 (0.37–0.75)	0.74 (0.48–1.15)
**Distance to hospital in an hour on foot**				
>2 h	79	76	1	1
<1 h	102	73	0.74 (0.48–1.15)	1.43 (0.78–2.63)
1–2 h	124	107	0.90 (0.60–1.35)	1.50 (0.92–2.44)

^*^p <0.05, ^**^p <0.01, ^***^p <0.001, AOR, adjusted odds ratio; CI, confidence interval.

## Discussion

This study examined self-referral practice and associated factors among women who gave birth in South Gondar zone primary hospitals. The prevalence of self-referral in this study was 46% with a 95% CI of 41.5% and 49.9%. This finding was consistent with a study conducted in Kenya (47.2%) (15) and the Pwani region, Tanzania (41.8%) (14).

However, the result of this study was lower than a study conducted in the Hadiya zone, Ethiopia (67%) (24). This difference might be due to the intervention that the Ethiopian health sector transformation plan focus on to improve the accessibility and quality of HCs since 2016 (43, 44). In addition, this might be also due to a variation in the study period, an increased number of health facilities/health professionals' every year, and advice given at ANC clinics. Furthermore, the observed lower self-referral practice might be a result of the government's ongoing efforts to improve maternal health service delivery.

The finding of this study was lower as compared with studies conducted in DCSH, Ethiopia (63.9%) (25), Western Ethiopia (82 and 84%) (4, 35), Indonesia (66%) (10), Nigeria (70%) (12), and Sudan (87%) (11) of outpatients who were self-referred. This difference might be due to a difference in the study population, which means labor is acute and progressive; women may not be able to walk as far as outpatients. In addition, our study specifically focuses on delivery, but the foregoing studies were on medical outpatients, which were non-specific.

As a result of our study, we also found lower findings than those reported by Tanzanian (75.4%) (27) and Nepalese studies (70.2% and 55%) (20, 23). This difference might be due to the intervention of health extension programs in Ethiopia, which is dynamic enough to shift tasks between HCs and the community (45). The other possible explanation could be due to computational variations. In our study, a mother was only considered to be self-referred if she bypassed health centers without having ANC or an appointment at the hospital. The difference with Nepal might also be due to the fact that their studies assessed bypassing referral hospitals, which have better quality and reputation than primary hospitals.

The finding of this study was different from a study conducted in Uganda (29%) (13). The possible explanation was that, unlike our study, the study in Uganda assessed self-referral regardless of the level of care. This implies that horizontal self-referral was unlikely because of service similarity. Additionally, this could be due to differences in the study area. Our study was conducted at the zonal level, which was wider than studies conducted in Uganda at the district level. This study was also higher than a study conducted in Ghana (33%) (17), Chris Hani Baragwaneth and KwaZulu-Natal studies in South Africa (19, 35 and 36%) (36, 46, 47), and a study conducted in India (37.7%) (26). This variation could be explained by the difference in quality health service availability and accessibility at lower PHCUs in Ghana, South Africa, and India, which are better than Ethiopia. This variation might also be due to a difference in the level of community awareness, access to information on the referral system, and health professionals' commitment. The variation with Ghana might be attributed to the fact that, in the Ghanaian context, data were collected within 6 weeks of the postpartum period when women came for post-natal care, which could be biased by women who did not attend post-natal care after delivery. Furthermore, the finding from Chris Hani Baragwaneth Hospital was determined with a small sample size (171) and a retrospective patient record review over five working days.

In this study, women who had no ANC visits and only 1–3 ANC visits were more likely to self-refer than those who had four and more ANC visits. The finding of this study was consistent with studies conducted in Nepal and South Africa (20, 36). This might be due to no ANC follow-ups or 1–3 ANC follow-ups which might indicate their poor awareness of the services given at HCs. Additionally, this might be due to the absence of birth preparedness and complication readiness plans, which ultimately influence the place of delivery. On the contrary, those who had full ANC might indicate their trust and dependency on the nearby HC for service and counseling, which might influence their decision on where to give birth. This implies that ANC follow-up has had a direct relationship with self-referral practice, and improving ANC coverage might reduce self-referral practice.

The findings of this study showed that women who had poor knowledge of the referral system were more likely to self-refer than those women who had good knowledge of the referral system. This finding was consistent with studies conducted in South Africa, Nigeria, and Ethiopia (4, 12, 25, 35, 46). First, this could be because knowledge is the key to increasing women's awareness of the general service provision of facilities and the chains of lower to higher healthcare facilities. Moreover, they are more likely to be aware of the existing referral system (48, 49). This difference might be due to a poor understanding of the cost of self-referral and the significance of the referral letter. This implies that the knowledge of the referral system has had a direct relationship with self-referral practice and improving client knowledge of the referral system might reduce self-referral practice.

In addition, women who used public transportation had higher odds of self-referral than those who traveled on foot. This finding is consistent with a study conducted in Western Ethiopia and Ghana (4, 17). The use of healthcare services may be facilitated by owning a means of transport (49). Due to the greater distance that is usually associated with bypassing facilities (50), it is more likely that bypassing will be accomplished by using a car or motorcycle and other public transportation than by walking (51). Therefore, our findings suggest that mothers who own and/or use their own cars/motorbikes or public transportation bypass their PHC facilities when seeking delivery service compared to those who lack such means of transportation and must walk to the healthcare facilities to receive care.

### Limitations and strengths

First, this study was a cross-sectional survey; this makes it impossible to draw causal inferences about the factors of self-referral practice. However, since most factors preceded the delivery, reverse causation is unlikely. This study might be prone to recall bias because some of the independent variables assessed the participant's previous experience. This may lead us to false results. To minimize these, they were asked to recite their recent experience.

## Conclusion

A facility-based survey conducted in SGZPHs found that nearly half of the deliveries were self-referrals. ANC follow-up, knowledge of the referral system, and mode of transportation to the hospital were factors significantly associated with the self-referral practice. It would have been good for the government to develop strategies to increase awareness and knowledge about ANC follow-up and referral systems among mothers. It is also necessary to establish monitoring and evaluation systems throughout health facilities for referral links. Furthermore, it would be better to conduct a study that takes into account the costs of self-referral practices in the future.

## Data availability statement

The original contributions presented in the study are included in the article/supplementary material, further inquiries can be directed to the corresponding author.

## Ethics statement

The studies involving human participants were reviewed and approved by the Institutional Review Board (IRB) of the University of Gondar, College of Medicine and Health Sciences, Institute of Public Health (Ref. No. IPH/2119/2014). The patients/participants provided their written informed consent to participate in this study.

## Author contributions

AE conceived the idea and contributed to the design, analysis, interpretation, report, and manuscript writing. TB, WN, DA, SM, and AK were involved in the design, analysis, interpretation of the data, and manuscript writing. All authors read and approved the final manuscript.
